# Anisotropic Gold Nanoparticles in Biomedical Applications

**DOI:** 10.3390/ijms19113385

**Published:** 2018-10-29

**Authors:** Claudia Kohout, Cristina Santi, Laura Polito

**Affiliations:** 1 Department of Chemistry, University of Milan, via C. Golgi 19, 20131 Milan, Italy; claudia.kohout@unimi.it (C.K.); cristina.santi@unimi.it (C.S.); 2 ISTM-CNR, Nanotechnology Lab., via G. Fantoli 16/15, 20138 Milan, Italy

**Keywords:** gold nanoparticles, anisotropic AuNPs, synthesis, biomedical applications

## Abstract

Gold nanoparticles (AuNPs) play a crucial role in the development of nanomedicine, principally due to their unique photophysical properties and high biocompatibility. The possibility to tune and customize the localized surface plasmon resonance (LSPR) toward near-infrared region by modulating the AuNP shape is one of the reasons for the huge widespread use of AuNPs. The controlled synthesis of no-symmetrical nanoparticles, named anisotropic, is an exciting goal achieved by the scientific community which explains the exponential increase of the number of publications related to the synthesis and use of such type of AuNPs. Even with such steps forward and the AuNP translation in clinic being done, some key issues are still remain and they are related to a reliable and scalable production, a full characterization, and to the development of nanotoxicology studies on the long run. In this review we highlight the very recent advances on the synthesis of the main classes of anisotropic AuNPs (nanorods, nanourchins and nanocages) and their use in the biomedical fields, in terms of diagnosis and therapeutics.

## 1. Introduction

**Figure 1:**
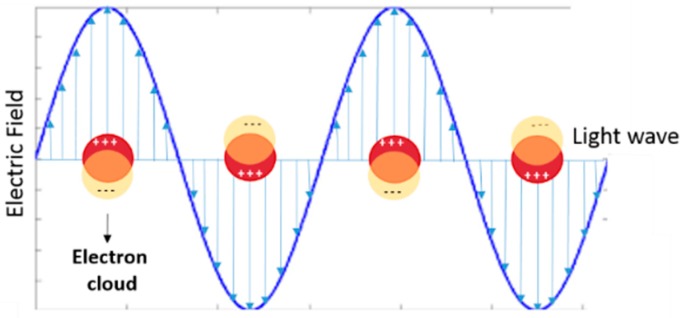
Graphical illustration of localized surface plasmon resonance (LSPR) band formation (in yellow circle the negative electron cloud, in red circle the positive electron cloud).

In recent years, the huge advances in the nanotechnology field have promised many breakthroughs in countless applications, in such a way that it is widely felt that nanotechnology will represent a new industrial revolution. Among the many types of material that can be easily manipulated at a size less than 100 nm, gold is one of the major players and its extraordinary features have been well known for millennia, when colloidal gold was used for decorative arts [1,2,3]. The deep red color of a nanometric solution of gold fascinated even the Egyptians during the 8th century BC, but the scientist who first realized that the color of colloidal gold was strictly connected to its interaction with the light, was Michael Faraday in 1857 [4], inspiring the theoretic work of Mie at the beginning of 1900 [5]. In his seminal work, Mie was able to explain the origin of the ruby color of a solution of spherical gold nanoparticles (AuNPs), describing the absorption and scattering of the light impinging on them and originating the surface plasmon band. Plasmons, better known as localized surface plasmon (LSP), result from the collective participation of free and easy polarizable electrons in noble metals and can be described as a negatively charged cloud excited by the interaction with the electromagnetic wave (Figure 1) [6,7]. As a consequence, metal particles with a suitable nanosize amplify the electromagnetic field near their surfaces and show an optical absorption spectrum with a maximum at the plasmon resonance frequency (LSPR) that for spherical gold nanoparticle of 10 nm in diameter is centered at 520 nm. The AuNP diameter and shape strongly affect the LSPR of the colloidal solution, shifting the band absorption in the visible and near infrared region (NIR). This kind of shift can be generated by the simple interaction among spherical nanoparticles (i.e., aggregation) or by the intrinsic anisotropic shape of the nanoparticles. When complex gold nanoparticles are used, Mie’s theory cannot predict their optical behavior and then different computational and mathematical methods are needed [8]. 

Due to the NIR shift of LSPR band, anisotropic AuNPs gain a huge interest from the scientific community in the last years, envisaging in their exploiting several new possible applications and improvements in biomedical fields [9,10]. The near infrared region is, in fact, the optimal optical window for nanomedicine application, as the absorbance from water and blood are limited, opening the way for non-invasive optical imaging and sensing modalities. The chance to fine modulate the LSPR spectrum of gold nanoparticles is the driving force which explains the significant number of papers related to the synthesis and applications of this kind of AuNPs. Beside the unique optical and electronic features, anisotropic AuNPs share with the isotropic ones a high biocompatibility and the tunable and reliable surface functionalization via thiol-chemistry. Suitable functionalized and water-dispersible anisotropic AuNPs have already been used in many bio applications. In this review, we will summarize the recent advances on the innovative use in nanomedicine, both in therapeutic and in diagnostic fields. Nevertheless, a fundamental role for the widespread and clinical translation of anisotropic nanoparticles is related to a reliable and reproducible high-quantity production of AuNPs. For this reason, in the first part of the review we will focus our attention on very recent advancement on nanoparticle production.

## 2. Synthesis

The two main approaches used to synthesize nanomaterials are the so called “bottom-up” or “top-down” protocols. The latter is related to the disruption of bulk materials to the nanometric size by means of physical or mechanical procedures such as laser lithography, etching or mechanical grinding. The “bottom-up” approach instead, is based on the building of the nanoparticles starting from molecular or atomic assembling; this is the most diffuse protocol for the synthesis of colloidal solution, as it can ensure a high quantity of material with a well-defined crystal structure. In general, the synthesis of spherical AuNPs is based on “bottom-up” procedure which envisage the reduction of Au^III^ ions in the presence of a suitable reducing agent such as NaBH_4_ or sodium citrate [1]. The synthesis of anisotropic gold nanoparticles in a colloidal solution is based on the “bottom-up” approach with a fine control of the face-selective crystal growth. The typical procedures to afford no-spherical AuNPs require the addition of specific surfactants (i.e., cetyl trimethylammonium bromide, CTAB) or the use of shape regulating agents, molecules able to cap or promote the growth of specific facet (i.e., silver nitrate). Although there is a large number of papers reporting on the synthesis of anisotropic gold nanoparticles, a general rule which regulates their synthesis is still missing [8,11].

**Figure 2:**
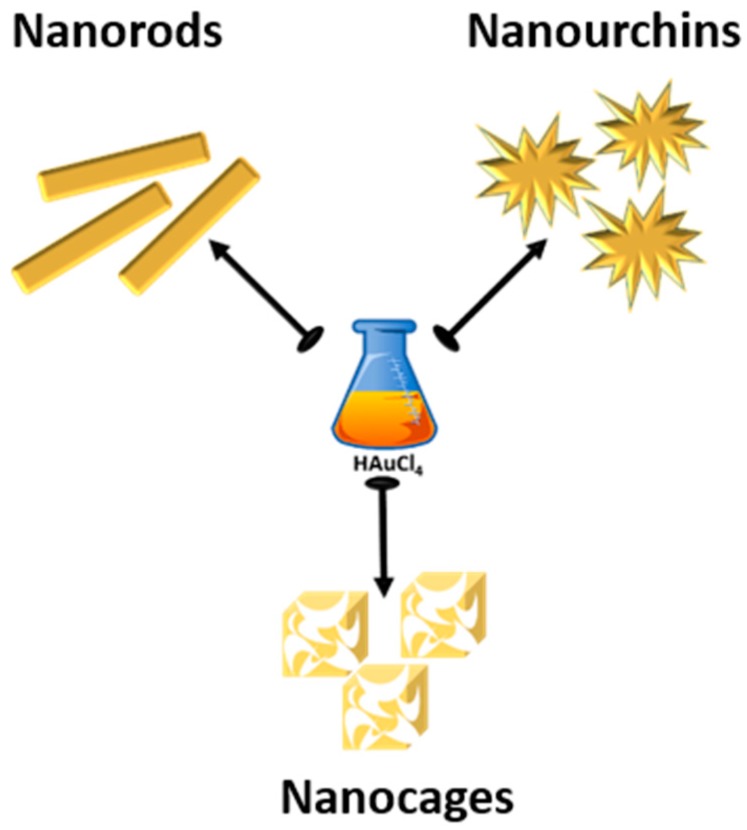
Anisotropic gold nanoparticles considered in the present review: nanorods, nanourchins and nanocages.

In the present manuscript we will review the very recent advances in the synthesis of gold nanorods (AuNRs), urchins-like gold nanoparticles (AuNUs) and gold nanocages (AuNCs) (Figure 2), obtained by means of a reduction of a tetrachloroauric aqueous solution. 

### 2.1. Synthesis of Gold Nanorods (AuNRs)

**Figure 3:**
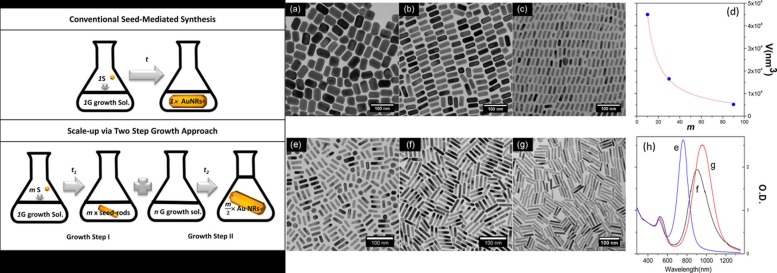
Left: Comparing the conventional seed-mediated protocol and the two steps approach. Right: TEM images, scale bars 100 nm. (**a**–**c**) Different AuNR volume in function of seed concentration. (**d**) Correlation between seed concentration scaling factor (m) and the final AuNR volume (V). (**e**–**g**) Effect of the change of surfactant in the second growth solution on the AuNR aspect ratio. (**h**) UV-vis spectra of the AuNRs e–g. Reproduced from [30] with permission from the American Chemical Society.

Gold nanorods represent the most diffused class of anisotropic shape gold nanoparticles [8,12,13]. The first synthesis was reported in 1997, when AuNRs were produced by an electrochemical reduction, in the presence of CTAB and characterized by UV-visible spectrometer [14]. The unique optical profile showed the two typical bands, and the authors demonstrated that the band at longer wavelength was tunable and dependent on nanorod’s ratio. This discovery was the beginning of a great interest toward this kind of material, and the seed-mediated protocol was the real starting point for the wide exploitation of AuNRs in many fields of applications [15,16,17]. The seed-mediated growth method is based on a two-step procedure. In the first step, a seed solution, containing spherical gold nanoparticles with a core diameter of 4 nm is prepared. The suitable CTAB surfactant is already present in the seed-solution, capping the small gold nanoparticles reduced to Au^0^ by sodium borohydride. Then, a named “growth-solution” is prepared by mixing CTAB, HAuCl_4_, a mild reducing agent (i.e., ascorbic acid) and silver nitrate (AgNO_3_). In the second step, a certain amount of the seed-solution is added to the growth-solution and a slow crystal growth is confirmed by a change in color, until the final formation of gold nanorods. This protocol proved to be very versatile and, additionally, methodological improvements have been done in order to obtain AuNRs with different aspect ratio and increased monodispersion [15,16,17,18], such as the use of a binary surfactant mixture (coupling CTAB with sodium oleate) [18], the introduction of microwave irradiation in the final stage of AuNR formation [19] or the use of pre-formed gold nanocubes as seeds [20]. Even if the protocol is widely diffused, some aspects that control the AuNR formation are not completely clarified (i.e., the temperature control, the pH and the silver nitrate role) and the growth behavior of gold nanorods is still a very hot topic. In order to predict and to have an improved control over AuNR synthesis and yields, different studies deeply investigated the mechanism of formation of this kind of anisotropic nanoparticles by mathematical modeling or using sophisticated techniques [13,21,22]. By using a population balance framework for mathematical modeling and numerical simulations, the authors were able to predict the final yield of a synthesis, introducing a new concept named “shape nucleation” which explains and predicts the extent of the presence of at least two morphologies, nanospheres and nanorods [21]. In the binary-surfactant protocol, the role of the temperature has been profoundly evaluated, giving a fundamental contribution on the comprehension of rod formation [23]. Among all the parameters, it is known that the addition of AgNO_3_ is a crucial step in many anisotropic gold nanoparticles synthesis to finely control the final shape formation. However, the role of silver nitrate in the AuNR formation is still unclear [24,25,26,27], and a key issue is related to the difficult of the reproducibility of high-quality AuNRs. Currently, the widely accepted mechanism relies on the preferential deposition of a silver monolayer on the Au {110} facet, therefore favoring the metal growth in the {100} direction, the so called underpotential deposition (UPD) [28]. A very recent contribution has correlated the lack of reproducibility to the inherent CTAB impurities [29]. By comparing different CTAB batches from different suppliers, the authors discovered that each batch contained different iodine quantities. Only modulating the silver nitrate addition on the base of this iodine amount, the reproducibility of AuNR was preserved. Therefore, they could demonstrate in a very elegant manner that the halide ions present in the mixture are a crucial factor in the AuNR synthesis mediated by silver nitrate, due to the high affinity of silver for anions. Vaia and collaborators explored a modified seed-mediated procedure in order to scale-up the AuNR synthesis maintaining the homogeneity, the reproducibility and the cost efficiency [30]. Their goal was obtained by developing a procedure based on a two steps synthesis (Figure 3a), increasing both seed and reagent concentrations. In the first step they synthesized a concentrated seed solution and added it to a diluted growth solution, to preserve the symmetry breaking which guarantee the product quality and purity. In the second step the seeds were added to a concentrated growth solution, affording the final AuNRs at a very high concentration and quality. Moreover, the authors demonstrated that their two-steps approach was feasible for controlling the final size and ratio of the nanorods. In particular, by increasing the seed’s concentration a decrement of the final nanorod volume was registered (Figure 3b-left). In the same time, by varying the surfactant mixture, a fine control on the nanorod ratio was achieved using as co-surfactant benzyldimethylhexadecylammonium chloride (BDAC). 

The change of CTAB as a soft-template agent with the less toxic dodecylethyldimethylammonium bromide (C_12_EDMAB) has been explored by Stone’s group [31]. The authors developed a modified seed-mediated protocol introducing, successfully, the new templating agent and showing that even the amount of C_12_EDMAB needed was higher than CTAB, the toxicity induced on Hep-2 and A549 cells was lower. The possibility to maintain the width of AuNRs less than 10 nm is particularly interesting, raising to a longitudinal plasmon resonance which can shift even about or over 1000 nm. Recently, the Murphy’s group explored the opportunity to set-up a scalable procedure to obtain mini gold nanorods with an aspect ratio (AR) ranging from 2.2. to 10.8 but with a width comprises within 10 nm [32]. The procedure is similar to the standard seed-mediated rod synthesis, and the major modifications are the use of a large volume of the seed solution and the low pH of the growth solution. Then, a further fine control over AR and yield has been achieved by tuning the concentrations of silver nitrate, hydrochloric acid and seed solutions. Moreover, to achieve AuNRs with AR between 5.6 and 10.8, ascorbic acid was successfully exchanged with hydroquinone (HQ), confirming the efficiency of this mild reductant already employed in the anisotropic gold nanoparticle synthesis [33]. Fixing the concentration of silver nitrate (0.36 mM), and varying the HCl amount added in the growth solution, the authors pointed out that the AR could increase only by lowering the pH of the solution. 

**Figure 4:**
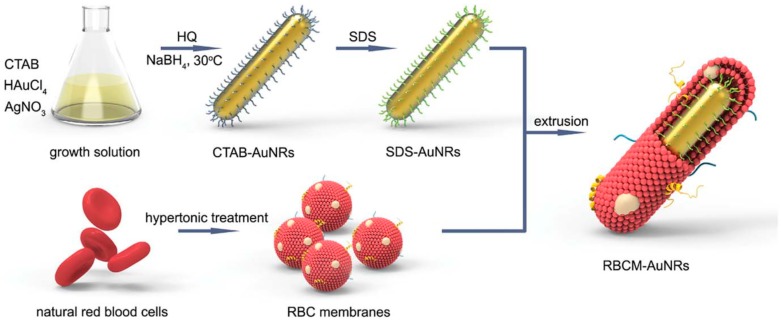
Schematic illustration of the production of the red blood cell membrane camouflaged gold nanorods (RBCM-AuNRs). Reproduced from [37] with permission from the Royal Chemical Society.

In 2005, Jana [34] proposed a different methodology for AuNR synthesis, based on a seedless protocol in order to obtain gram quantities of monodisperse AuNRs. The addition of the strong reducing agent (NaBH_4_) was introduced in the growth solution, so far, the in-situ formation of small AuNPs acted as seeds, promoting the formation of CTAB capped AuNRs. Since this first contribution, a large number of papers based on the seedless protocol have been published, in order to increase the efficiency of AuNR synthesis, enhance their stability, modulate the aspect ratio, eliminate or decrease the CTAB concentration (see recent reviews [32,35]). Zubarev and colleagues [36] demonstrated that a well-known additive as poly(vinylpyrrolidone (PVP), extensively employed as stabilizing and reducing agent for many kind of metal nanoparticles, could be efficiently used to reduce the AuNR size in a seedless protocol. For the first time, PVP added in very low concentrations with respect to Au^3+^, was used to control the shape growth of gold nanorods in the presence of CTAB, acting as a stabilizing and not as a reducing agent. One of the major drawbacks that limit the clinical translations of gold nanorods is the cytotoxicity of CTAB. Therefore, any improvement in AuNR synthesis which can limit the use of the templating agent or that permits a reliable ligand exchange is very desirable. Recently, a modified seedless procedure has been developed and coupled with an interesting protocol to camouflage the gold nanorods with the red blood cell membrane (Figure 4) [37]. The synthesis is based on the use of the mild reductant hydroquinone, already known as additive agents in the synthesis of anisotropic gold nanoparticles [33]. Controlling the amount of hydroquinone and CTAB a fine control on the AuNR size was possible and, by using sodium dodecyl sulfate (SDS), an effective removal of CTAB was achieved. Then, the final extrusion of a mixture containing SDS-coated AuNRs and the red blood cell membranes afforded an innovative and promising core-shell nanostructure, active as biomimetic theranostic material. 

**Figure 5:**
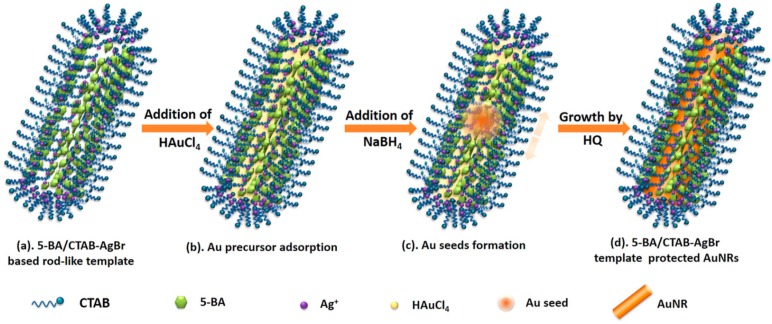
Scheme of seedless synthesis technique based on the use of 5-bromosalicylic acid and hydroquinone. Reproduced from [38] with permission from the John Wiley and Sons.

The use of HQ has been exploited for the seedless modified protocol proposed by Wang in 2017 [38]. The authors introduced two main modifications: the introduction of 5-bromosalicylic acid (5-BA) as template regulator at the beginning of the micellar formation and the addition of HQ as weak reducing agent during the growth phase (Figure 5). The 5-BA intercalate within the CTAB layer and, subsequently, the phenyl ring and the carboxylate could modulate the packaging density of micelle layer, driving the final morphology. The authors conducted a systematical work, finding out that in diluted CTAB conditions (50 mM), the ideal 5BA/CTAB ratio to obtain the best AuNRs in terms of efficiency and purities (i.e., no spherical sub-products) was between 0.1 and 0.25. To improve the reproducibility of the protocol, the authors had to modulate the nucleation process introducing a mild reducing agent after the addition of NaBH_4_. HQ resulted to be the best choice and its concentration was fixed within 1 and 5 mM in order to have the best high-quality AuNRs. With the correct Au/CTAB ratio and pH, and introducing the modifications proposed, the new seedless protocol seems to be very useful to obtain high quantity of reproducible and monodispersed AuNRs. 

Very recently, AuNRs have been given green and biologically available curcumin in combination with a template supramolecular agent, the curcubit[7]uril [39]. Curcumin was able to reduce Au^III^ to Au^0^, forming spherical AuNPs that, in the presence of the host molecule, shaped the NP into selected nanorods. 

### 2.2. Synthesis of Gold Nanourchins (AuNUs)

The synthesis of multibranched AuNUs, accomplished in a reliable and reproducible way, is more challenging respect to the results achieved for AuNRs. In general, AuNUs can be obtained by means of two different approaches: the seed-mediated growth assisted by the presence of a capping agent and the one-pot aggregation of many seeds, followed by their growth. The first synthesis was published by Carrol and collaborators in 2003 [40], affording a mixture of monopod, bipods, tripods, and tetrapods. The protocol was based on HAuCl_4_ reduction mediated by ascorbic acid (AA) as reducing agent, sodium hydroxide, CTAB and promoted by the presence of silver nanoplates. Successively, multibranched AuNPs were synthesized by Murphy and co-workers by a modification of the seed-mediated protocol for the synthesis of AuNRs [41]. As already demonstrated for AuNR synthesis, surfactants such as CTAB or sodium dodecyl sulfate (SDS) and additives such as silver nitrate played a central role in promoting and driving the anisotropic growth of gold nanocrystals. The reductive agent is crucial as well, and different molecules have been used to induce the formation of multibranched, such as hydroxylamine or hydroxylamine sulfate or N,N-dimethyl formamide (DMF) [41,42,43]. With the latter protocol, developed by Liz-Marzán and collaborators, a fine regulation of the length of arms could be attempted, tuning the arm size from 45 to 160 nm. Nevertheless, a control over the number or urchins is still very difficult to achieve [44]. Recently, the growth mechanism of the tips in a multibranched nanostructure has been studied by an insight elucidation of a seed-mediated protocol [45]. The employed strategy was based on the use of DMF as mild reducing agent which promoted the reduction of Au^III^ to Au^0^ and PVP which acted as further reductant and stabilizing agent. The authors were able to follow the formation of the tips since their first protrusion, correlating the length of the tips with the concentration of seed solution and the time of the reaction. At the lower seed concentration, longer branches formed while an increasing in the central core was observed at later stage of growth.

In the one-pot synthesis, different reducing agents have been exploited such as ascorbic acid, sodium citrate and H_2_O_2_, or N-2-hydroxyethylpiperazine-N-2-ethanesulfonic acid (HEPES). With these protocols, a control over the homogeneity of the multibranched NPs is very challenging, and in many cases the final nanoparticles are depicted as a first aggregation followed by growth of multiple seeds. These kinds of morphology are often addressed as “sea urchin”, “nanoflower”, or “raspberry”. A control of the spike lengths in the synthesis of AuNUs has been performed by exploiting a template medium based on the use of Triton X-100 (TX-100) that form a lyotropic liquid-crystalline (LLC) phase when mixed with water [46]. TX-100 and water in the optimal ratio 42/58 wt% show a hexagonal columnar LLC phase, are able to drive and constrain the formation of long spikes. Above 29 °C, the LLC phase changes into a micellar phase contributing to the formation of nanourchins described more as nanoflowers. An improved protocol to afford a well-defined gold nanostars comprised of 4 to 6 branches has been developed by Pallavicini’s group. The authors based their synthesis on a seed-mediated approach in which, during the growing stage, the TX-100 is added as surfactant, affording multibranched gold nanoparticles with a tip to tip distance of 75 nm [47]. Recently, the authors modified this protocol, increasing the seed/growth volume ratio from 0.012/10 to 0.055/10 to shrink the overall dimension of the nanostars, affording gold nanoparticles with a relative diameter of 45 nm and a consequent blue-shift of their NIR-LSPR [48]. In order to have a procedure reliable and reproducible, the Vo-Dinh group has developed in 2012 a seed-mediated protocol based on the absence of any surfactant [49]. The procedure afforded multibranched AuNUs by adding AgNO_3_ and ascorbic acid to a mixture of AuNP seeds and HAuCl_4_, but the nanoparticles obtained were not homogenous. Confirming the importance of a surfactant-free procedure, the protocol was very recently modified [50], pointing out the crucial steps which could be responsible for the polydispersion and poor reproducibility. First of all, the addition of silver nitrate was anticipated respect to ascorbic acid, ensuring a major homogeneity of the final sample. Moreover, the ratio of Au^3+^ and Ag^+^, the controlled pH and the halide content of the mixture turned out to be important point for the production of AuNUs and for the number and length of branches.

**Figure 6:**
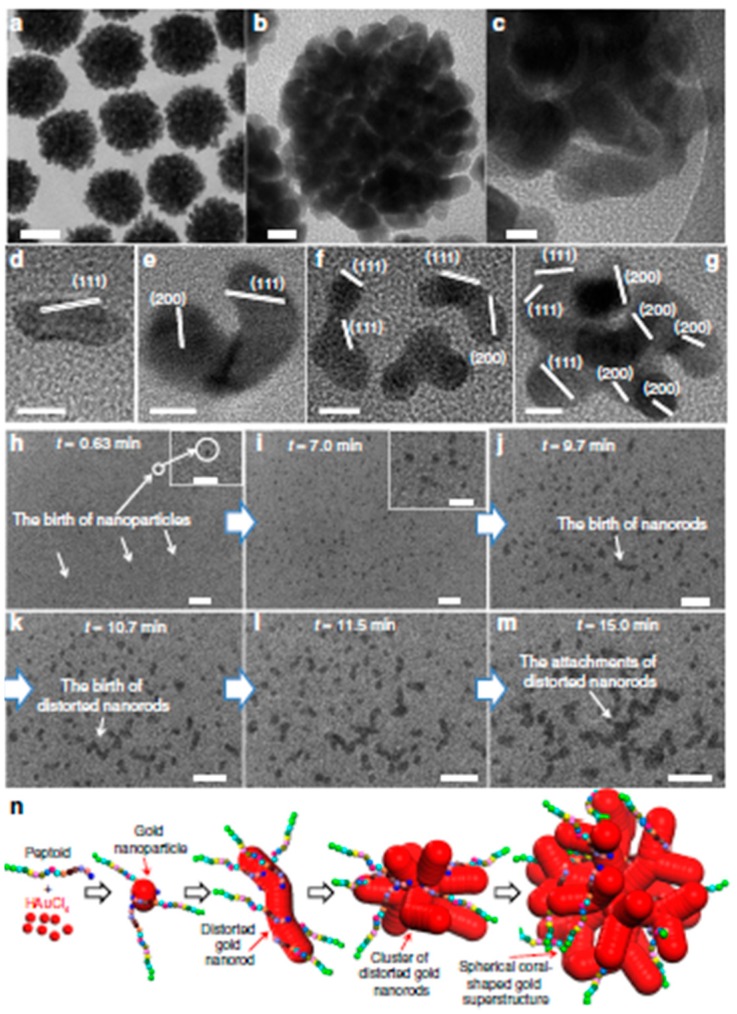
(**a**,**b**) TEM images of the coral-shaped nanoparticles (scale bar; (**a**) 50 nm; (**b**) 10 nm). (**c**) HR-TEM (scale bar, 5.0 nm). (**d**–**g**) HR-TEM images of distorted gold nanorods and clusters in early stages of coral-shaped particle formation (scale bar, 5.0 nm). (**h**–**m**) Sequence of in situ liquid cell TEM time (scale bar, 20 nm) showing the early stages of coral-shaped particle formation (insets of h, i are the magnification of TEM images, scale bar at 10 nm). (**n**) Schematic representation for the multibranched formation of gold nanoparticles induced by peptoids. Reproduced from [51] with permission from the Nature Publisher.

Taking inspiration from natures, a control over gold nanourchins synthesis has been obtained by designing and synthesizing poly-*N*-substituted glycines, named peptoids, characterized by a high biocompatibility, stability and a defined chemical sequence [51]. Having in mind the numbers of medical applications in which AuNUs could give great advantages, new synthetic protocols which avoid toxic surfactant are highly desirable. For the first time, a set of peptide mimic has been designed in order to synthesize gold nanoparticles with a defined and predictable shape, from spherical to highly branched (Figure 6). At the beginning of the synthesis the peptoids are able to form distorted AuNRs which can randomly strongly aggregate, affording the hyperbranched final nanoparticles. To get this important result, the peptoids should incorporate specific groups such as *N*-(2-carboxyethl) glycine, *N*-(4-aminobutyl)glycine, *N*-(4-aminoethyl)glycine), and *N*-[2-(4-X-phenyl)ethyl]glycines] which ensure a final molecules with an amino side chain, strong hydrophobicity and a defined arrangement.

Another type of seedless protocol has been developed by using, in a one-pot reaction, bovine serum albumin (BSA) protein as template agent and ascorbic acid as reductant [52]. Albumin is a well-known protein present in the blood serum with an isoelectric point (pI) at 4.7. When mixed with HAuCl_4_ at a pH below its pI, BSA is predominantly protonated and AuCl_4_^−^ ions can easily adsorb on the protein surface. The successive addition of the reducing agent induces the reduction of Au^3+^ to Au^0^, by affording anisotropic gold nanoparticles whose morphology is controlled by the BSA shape. In fact, at pH 2.5, BSA assumes an expanded conformation with a loss of the intradomain helices which let the electrostatic adsorption of gold anions and the consequent formation of multibranched AuNUs with a LSPR centered at 808 nm. Another one-pot procedure has been developed by exploiting the reducing ability of a water-soluble polymer, the pyromellitic dianhydride-p-phenylene diamine (PPDDs), at room temperature [53]. By using a low concentration of gold salts (800 μL, 0.391 mM) in 2 mL of PPDDs, the polymer is able to reduce the gold precursor, by inducing an anisotropic shape affording multibranched nanoflowers with urchins long up to 26 nm and an overall nanoparticle size comprises between 70 and 120 nm. A change of HAuCl_4_ concentration, temperature and PPDDs/gold ratio justify a modification in nanoparticle shape giving the opportunity to tune to isotropic spheres or to nanourchins with modulated tips. The increasing interest towards the synthesis of multibranched gold nanoparticles is tested by continuously improvements in their manufacturing and applications. The impressive enhancement of SERS signals in the presence of gold nanostars (AuNSs) or nanourchins induced many groups to study new synthetic ways to obtain these structures with these finalities. Recently, in order to produce polydimethylsiloxane (PDMS) material functionalized with AuNSs on the surface, the ability of PDMS to reduce metal ions in situ has been exploited [54]. It was already known that the residual presence of the curing agents containing Si-H groups let the polymerized material act as reductive agents able to reduce gold (III) to gold (0) [55], but for the first time, this ability was exploited to produce a controlled film of multibranched AuNSs, directly on the PDMS surface. After PDMS polymerization, a small quantity of HAuCl_4_, sodium citrate and hydroxylammonium chloride have been dropped on the surface and the metal reduction was checked in a time frame of 20–300 s. The result was so successful that a SERS experiment to check the presence of a pesticide on an apple skin could be performed.

**Figure 7:**
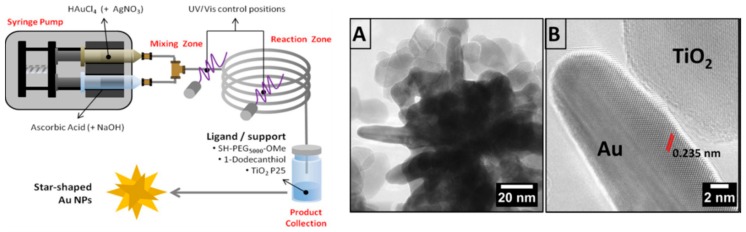
Left: Scheme of the fluidic production of multibranched AuNUs. The precursors loaded in the syringes can be mixed in the T-mizer (mixing zone), in the reactor (1 m) the AuNUs form and drop directly in an excess of ligand for proper stabilization. Right: (**A**) TEM image of a single multibranched gold nanoparticle immobilized on titania; (**B**) HR-TEM image of a single branch interacting with a titania. Reproduced from [56] with permission from John Wiley and Sons.

One of the major drawbacks which hamper the diffusion of anisotropic nanoparticles in wider application is strictly connected to the difficult in their reproducibility. Each single batch can be different from the others as too many parameters control the morphology, size and stability of NPs. In order to overcome these issues a microfluidic protocol was developed to produce, for the first time, multibranched gold nanoparticles at room temperature and avoiding the use of toxic template (Figure 7) [56]. The methodology is based on the accurate mixing of gold precursor (HAuCl_4_) and ascorbic acid as reducing agent, in a proper ratio and with a flux fixed at 5 mL/min, while the fine control of the morphology was achieved by adding silver nitrate. The great advantage produced by this protocol is the chance to synthesize, in continuous, high quantity of AuNUs which can be directly functionalized by only dropping the resulting NPs in an excess of stabilizing ligand or solid support such as SiO_2_ and TiO_2_ (Figure 7A,B).

### 2.3. Synthesis of Gold Nanocages (AuNCs)

**Figure 8:**
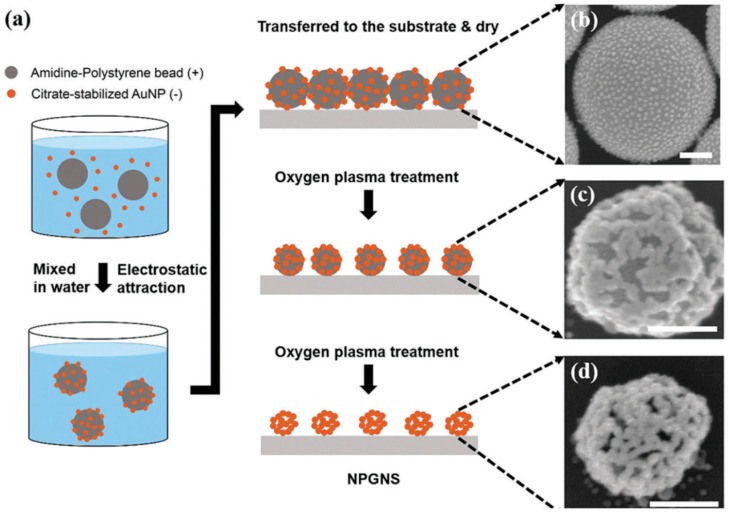
(**a**) Procedure for the synthesis of AuNCs by starting form polysterene beads. (**b**–**d**) SEM images of each intermediate step (scale bar 200 nm). Reproduced from [65] with permission from Elsevier.

There is another class of anisotropic gold-based nanostructure that exhibits tunable LSPR properties, the gold nanocages (AuNCs), which include nanostructures with hollow interiors or porous walls. Besides their optical features, these nanoparticles gain ever increasing interest in biomedical fields due to their cavity and, therefore, opportunity to load and deliver drugs or theranostic agents [57]. Their synthesis is generally based on the galvanic replacement of silver nanoparticles used as sacrificial template [58]. To synthesize uniform AuNCs it is necessary to manufacture well-monodispersed silver nanoparticles, remembering that the size of the pore is due to the HAuCl_4_/Ag ratio. By improving the control of the silver nanoparticles synthesis, ultrasmall silver templates have been produced (9 nm) affording successively AuNCs with a very thin gold shell which turned out in a LSPR band ranging from 600 to 900 nm [59]. Other typical protocols to synthesize AuNcs are based on the selective removal of silver from an Au-Ag alloy by using strong acidic solution [60,61] or the self-assembly and fusion of gold nanoparticles in suspension [62,63]. Recently, the synthesis of gold nanocages has been performed by an innovative use of lanreotide acetate (Lan), the salt of a synthetic cyclic octapeptide [64]. Lan is well known as an anti-cancer agent, able to address tumors by binding somatostatin receptors. In water solution, Lan can self-assemble and present free amine groups and a disulfide bond, inclined to graft gold surfaces. The authors exploited the features of Lan, achieving both gold nanocages and gold nanoshells (AuNShs) through incubation, at different pH, Lan with HAuCl_4_. In particular, at pH 2, gold nanocages was synthesized while, working at pH 6, a more uniform gold shell on the Lan nanostructure was afforded. The so-prepared AuNCs has been successfully employed in photothermal applications. Another technique recently employed to obtain AuNCs has been developed by Kim and collaborators [65]. Avoiding the dealloying method which foresees the removal of silver by using strong acid solutions, the authors proposed the use of a controlled plasma etching to afford gold nanocages. The new protocol is based on the confinement in a discrete space (a polystyrene bead) of a fix number of citrate-capped AuNPs (Figure 8). In this way the grafting agent can be easily removed by plasma etching, favoring a controlled sintering of AuNCs gold nanoaggregates on the polymer particle, followed by the final removal by oxygen plasma of the polystyrene core to afford a hollow cage gold nanostructure. 

To obtain a robust nanocage, decreasing in the same time the amount of silver in the nanostructure, a procedure based on three steps has been developed [66]. At the beginning a shell of gold and silver alloy is formed by galvanic replacement on silver nanoparticles template. Then, a gold layer was reduced on the outer shell by means of ascorbic acid to render stronger the structure, tuning the shell thickness from 7.4 to 10.4 nm. The final step corresponded to the dealloying process by means of HAuCl_4_ addition, which dissolves the silver atoms from the alloy shell, affording a final gold nanocage (average diameter centered at 80 nm) with a lower amount of silver respect to other synthetic protocols. These AuNCs have been used as photothermal agent, exhibiting a conservative LSPR peak centered at 840 nm. Such a result is particularly important when compared with analogue silver/gold alloy nanostructures which in in vivo and in vitro test collapse for the loss of Ag^+^ ions.

**Figure 9:**
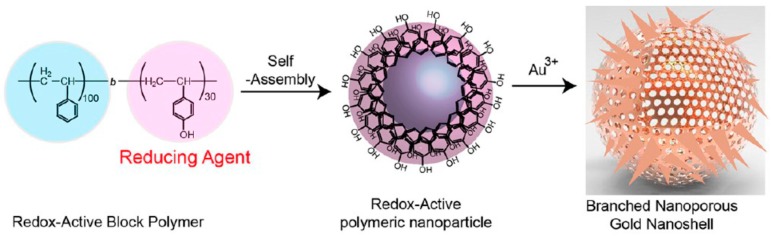
Schematic representation of the production of multibranched-hollow gold nanoparticles, based on self-assembled polymer template. Reproduced from [67] with permission from the American Chemical Society.

Even if nanocages have many advantages in terms of LSPR and cavity, the challenge is to combine the anisotropic shape with the hollow interior. This goal has been achieved [67] by using as template core a diblock polymer poly(vinylphenol)-b-(styrene) (PVPH-b-PS), that self-assembled into nanoparticles of 60 nm in diameter. The author exploited the reductive properties of the phenols exposed on the surface of the polymeric NPs to reduce Au^III^, affording a gold multibranched nanoshell (Figure 9).

The use of selected organic molecules exposed on the surface of NPs to reduce gold have been used even for the synthesis of other hybrid NPs [68], and has the great advantage to realize the reduction of gold in one-step, without the need to prepare seeds. The final step of this protocol is based on the dissolution of the polymeric template by using an organic solvent as DMF or THF, affording an exciting hollow/multibranched gold nanostructures. The resulted AuNCs have a LSPR band red-shifted respect to other anisotropic nanostructures, and have been used as real multimodal nanoagents carrying a Raman tag able to track the drug release activated by a laser irradiation.

## 3. Anisotropic Gold Nanoparticles in Bio-Sensing

### 3.1. Surface Enhanced Raman Scattering (SERS)

**Figure 10:**
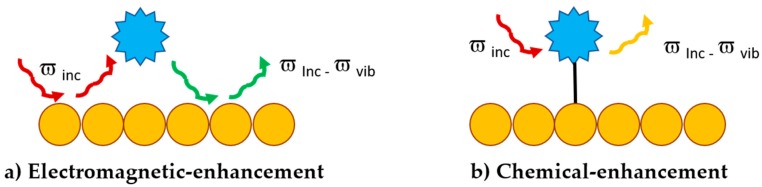
Two mechanisms at the base of the Raman signal enhancement: (**a**) electromagnetic and (**b**) chemical.

Raman spectroscopy is a vibrational spectroscopic technique based on the inelastic scattering of photons by the analyte molecules. The changes in the scattered photon frequency are proportional to the difference in vibrational energy levels of the molecule. However, only a small fraction of incident photons shows inelastic scattering and the Raman signal is extremely weak [69,70]. McQuillan and collaborators observed for the first time a strong enhancement in the intensity of the pyridine Raman signal, when dropped on a rough silver electrode [71]. In the following years, strong Raman signal enhancements were observed when molecules were grafted onto metallic nanostructure surfaces, named “SERS-active substrates” [72]. The mechanism by which this enhancement takes place can be explained by two main theories: the electromagnetic [73] and the chemical enhancement [74] (Figure 10).

When the incident light has the same resonance frequency of the excitation of LSPR band of the metallic nanoparticles, the generation of localized dipoles leads to an enhancement in the electric field around the nanostructure. The enhanced electric field interacts with the sample molecule and results in an induced dipole in the analyte and therefore an enhancement in the Raman signal (Figure 10a). This phenomenon is known as electromagnetic-enhancement and the corresponding enhancement factor is around 10^10^–10^12^. The chemical enhancement involves a charge-transfer mechanism between the metallic nanoparticle and the analyte molecule; it is necessary to have a close contact between the two elements in order to observe the enhancement. The chemisorption of the molecule on the metallic surface, leads to the generation of new electronic states with intermediate energies, resonating with the resonance frequency of the metal. Exchange of charge between analyte and substrate can take place in resonance conditions and results in the chemical-enhancement of Raman signal (Figure 10b). The enhancement factor obtained with this mechanism is around 10^3^–10^5^. In any case, to observe an enhancement of the Raman signals, the analyte molecule must be linked or close to a nanostructured metallic substrate, commonly noble metals, since they do no present any Raman active mode. To evaluate the enhancement of the signal obtained respect to the traditional Raman spectroscopy, the parameter widely used is the average enhancement factor (EF), [75] calculated as follow (Equation (1)):

(1) $$EF=\left(\frac{I_{SERS}}{N_{Sinf}}\right)/\left(\frac{I_{R}}{N_{Vol}}\right)$$

where *I_SERS_* and *I_R_* are the intensities of a representative peak in SERS and Raman spectra, *N_Surf_* and *N_Vol_* are the average number of the adsorbed molecules in the scattering volume for SERS and for Raman experiments. A simplified formula (Equation (2)) is often used to calculate the analytical *EF*, when the spectra are collected under identical experimental conditions:

(2) $$AEF=\left(\frac{I_{SERS}}{C_{SERS}}\right)/\left(\frac{I_{RS}}{C_{RS}}\right)$$

where *C_SERS_* and *C_RS_* are the concentrations of analyte in solution. The high sensitivity and chemical specificity of SERS make the technique suitable and interesting for a wide variety of applications in biomedical fields. In this contest, the use of anisotropic gold nanoparticles has several advantages with respect to isotropic nanoparticles as, due to the branched morphology they display “hot points” characterized by large electromagnetic fields.

One of the major roles of sensors in nanomedicine is the detection of biomarkers, to speed up the diagnosis processes. Ou et al. [76] exploited the multiplexing SERS experiments for in vivo detection of the immunomarker programmed death ligand 1 (PD-L1) and of the epidermal growth factor receptor (EGFR) in breast cancer tumors. PD-L1 is upregulated in various tumors and inhibit T-cell proliferation, leading to immunosuppression and its overexpression is correlated to the EGFR upregulation, which promotes tumor progression. SERS showed to be a highly convenient method for the simultaneous detection of PD-L1 and EGFR because the technique enables a multiplexed detection, thanks to narrow line widths of the vibrational signature of different Raman tags. The multiplexing tests were based on the synthesis of a set of urchinated gold nanoparticles (AuNUs) functionalized with two different Raman tags (2-nitrobenzoic acid and 5,5′-dithiobis (2-nitrobenzoic acid)) and further decorated with antibodies (anti-PDL1 or anti-EGFR) able to select the markers directly in vivo. The detection of a very important cancer biomarker, the protein kinase activity (PKA), has been recently achieved by the development of a AuNU-based SERS substrate and functionalized with bovine serum albumin-Kemptide (AuNU-BSA-Kem) [77]. Kem was conjugated to BSA by using the carbodiimide crosslinker chemistry and, then, BSA-Kem was non-specifically adsorbed on AuNU surface. To detect the PKA activity the authors used the principle component analysis (PCA) which is based on the isolation of two significative peaks in Raman spectra, at 725 and 1395 cm^−1^. The ratio between the intensities of these two peaks represents the extension of Kem phosphorylation and, consequently, the fundamental measure of the PKA activity. Inflammatory biomarkers are useful signals for disease diagnoses, and therefore, reliable and sensible tests to detect their presence earlier is highly desirable. SERS probe nanorods containing 4-mercaptobenzoic acid (4-MBA) as Raman reporter were tested by Hattori’s group [78] for detection of intracellular adhesion molecule-1 (ICAM-1), an inflammatory biomarker in macrophages. AuNR/4MBA@Anti-ICAM-1 sensors were incubated in murine macrophage cells, used as model line. The cells were stimulated with lipopolysaccharide (LPS) and SERS measurements were performed at different time-points after LPS addition. In contrast with traditional methods such as enzyme-linked immunosorbent assay (ELISA) or fluorescent-labelling techniques, ICAM-1 molecules could be detected only one hour after LPS treatment, instead of the common 5 h, confirming the high potential of these SERS probes. Considering that one of the most important features of new sensor devices is the response rate and an easy accessibility, an inexpensive paper-based lateral flow strips (PLFS) for the detection of neuron specific enolase (NSE) has been developed. By combining AuNUs with a Raman tag and silica, an effective system to detect the presence of NSE, a biomarker of traumatic brain injury, has been produced [79]. This device provides higher sensitivity and lower limit of detection (LOD) in blood plasma-containing matrix, compared to traditional colorimetric PLFS.

**Figure 11:**
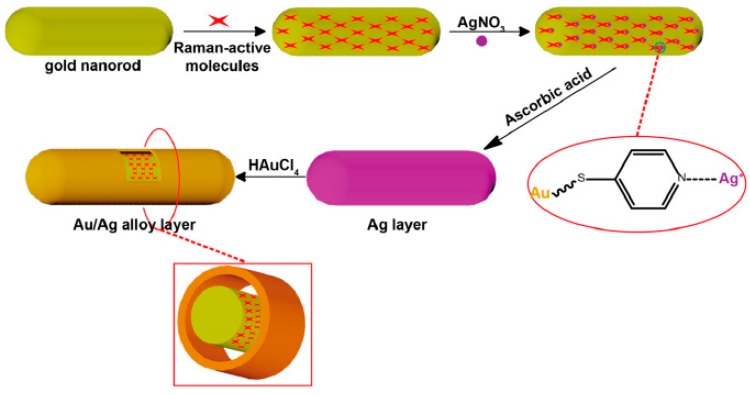
Schematic process for Au-AgAu core-shell structures synthesis. Reproduced from [80] with permission from the American Chemical Society.

Besides biomarker identifications, circulating tumor cells could be detected directly by using smart core-shell plasmonic nanorods [80]. To achieve this goal, Au nanorods were firstly decorated with Raman reporters that present a great affinity for Ag^+^ ions (e.g., 4-mercaptopyridine). In this way, a new uniform nucleation site was created to form a sacrificial Ag shell. The Au-AgAu core-shell structure was then obtained through galvanic replacement of the Ag shell (Figure 11). With this kind of nanosystem, the encapsulated Raman reporter works as internal standard for quantitative SERS analysis. The further surface functionalization of these structures with specific aptamers to circulating tumor cells (MCF-7) allowed for the detection of a minimum number of 20 cells in a blood mimicking fluid, making this system very interesting and promising for biomedical sensing applications.

The detection of biochemical compounds plays a fundamental role not only in early recognition of diseases but even in the control of mesenchymal cell differentiation. In this contest, recently, Cao et al. [81] developed a high-performance in vitro sensing platform to monitor the biochemical composition changes during bone marrow mesenchymal stem cells (BMSCs) differentiation. As it is known, BMSCs can differentiate in different cell types, including osteocytes and adipocytes, but they can also convert into cancer-associated fibroblasts and have a crucial role in tumor progression. To produce the SERS-based sensors for the non-invasive identification and discrimination of BMSCs, silicon wafer functionalized with anisotropic hollow Au nanoflowers (HAuNFs) have been developed. Firstly, HAuNFs were prepared by using hydroquinone and sodium citrate as reducing agents, through a seed-mediated growth approach. Then, hydroxyl silicon wafers were dipped in poly-(diallyldimethylammonium chloride) (PDDA) solutions, followed by immersion in the HAuNF solution. HAuNF-decorated wafers were then incubated with BMSCs. Treatments with adipogenic and osteogenic inductors were performed to obtain the differentiation of the cells into adipocytes and osteocytes respectively. By monitoring the differentiation with SERS, it was possible to detect an increase in protein, nucleic acid and lipid components during the BMSC differentiation, together with a decrease in amino acids and carbohydrates, with high reproducibility and good sensibility.

The high impact that gold-based nanomaterials could have on daily human health can be verified by the studies developed to detect viruses or antibiotic resistance bacteria. Recently, AuNUs have been developed with the final goal to early detect the presence of human enterovirus, the EV71, in biological media [82]. This enterovirus can provoke the quite diffuse hand, foot and mouth disease and it is associated with neurologic and systemic complications. The nanosensors, based on AuNUs conjugated to recombinant scavenger receptor class B member 2 protein (SCARB2) for EV71 detection, spontaneously aggregate when dispersed in biological media, affording strong Raman peaks at 390, 510, 670 and 910 cm^−1^. In the presence of EV71, the enterovirus can bind the functionalized anisotropic gold nanoparticles, preventing their aggregation, as confirmed by the disappearance of the Raman peaks at 510, 670, 910 cm^−1^ and the decrease of the peak intensity at 390 cm^−1^. This method, after minimal sample preparation, allows to detect AV71 in protein-rich biological medium with high sensitivity (107 pfu/mL). On the other hand, the antibiotic resistance is an increasingly widespread phenomenon and a challenge for the public health. One of the current problems is associated with Carbapenem-resistant Enterobacteriacae (CRE), therefore it is extremely important to develop fast methods for antibiotic activity detection. Kang et al. [83] developed, as a proof-of-concept, a rapid and semi-quantitative method for in patient-detection of carbapenemase activity. The SERS-based strategy allowed for obtaining results after 25 min, a great result compared to the classical protocols that, usually, require hours or even days. The method is based on the direct functionalization of AuNS with ceftriazone, a β-lactam antibiotic containing available amino groups which can coordinate the gold surface. In the presence of *E. coli* bacteria that do not present antibiotic resistance, SERS spectra changed due to the β-lactam ring cleavage, registering a decrease of the peak intensity at 1358 and 1495 cm^−1^. The authors interestingly demonstrated that, in principle, it is possible to quickly detect the antibiotic activity and to identify the antibiotic-resistant bacteria.

### 3.2. Colorimetry

**Figure 12:**
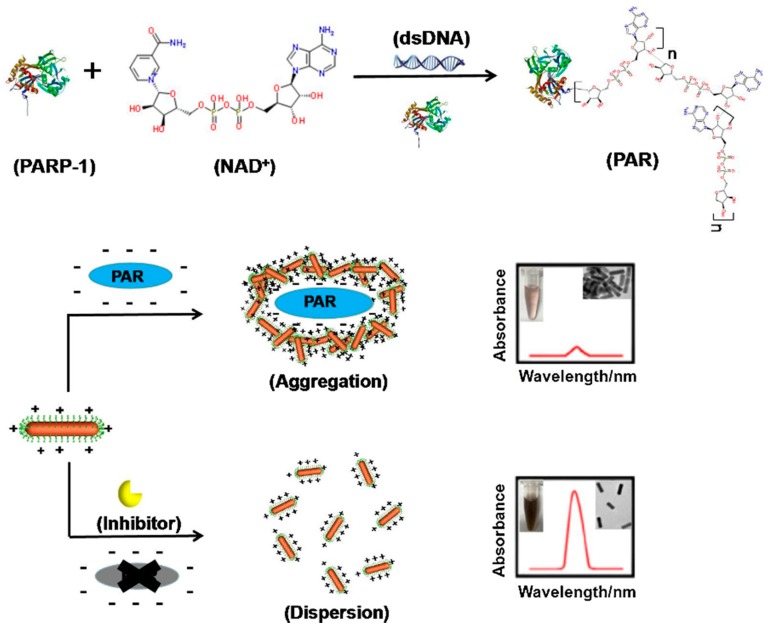
Up: scheme of the formation of poly(ADP-ribose)(PAR), catalyzed by poly(ADP-ribose) polymerase (PARP-1). Down: Scheme of the colorimetric strategy based on AuNRs without and with PARP-1 detector inhibitor. Reproduced from [87] with permission from Elsevier.

Sensors based on colorimetry have great advantages since they are cost-effective and the reading-out is fast and, in principle, can be gained in absence of instruments, since most of the color changes are visible with naked eyes. These sensors exploit the correlation between the NP concentration in solution and their absorption wavelength [84], and the absorption (color) changes taking place when aggregation or dispersion events occur. Colorimetry-based sensors are versatile as any analyte that has a direct or indirect role in particle aggregation or de-aggregation can be detected [85,86]. In this contest, a counterion-induced AuNRs aggregation method for poly(ADP-ribose) polymerase (PARP-1) detection has been developed [87]. PARP is a superfamily of enzymes particularly interesting as their levels are associated with various types of cancer. Since the active PARP-1 metabolize NAD^+^ into nicotinamide and ADP-ribose, a large part of the existing protocols for PARP-1 detection are based on NAD^+^ concentration measures but, these methods suffer of not optimal limit of detections [88]. On the contrary, the sensors developed by Wu (Figure 12) are based on the aggregation of CTAB-AuNRs, induced and correlated by poly-(ADP ribose) (PAR) concentration. CTAB confers to AuNRs stability and a strong positive charge (ζ-potential = 36.7 mV). When PARP-1 is incubated with NAD^+^ and activated DNA, the negatively charged PAR adduct is achieved. The presence of negative charged molecules induces the aggregation of AuNRs (characterized by a positive ζ-potential), leading to a change in the absorbance, in the color and in ζ-potential. With this method, also PARP-1 inhibitors activity can be easily measured.

By using a colorimetric approach, a semi-quantitative method for H5N1 virus analysis was developed by Xu et al. [89], exploiting highly uniform gold nanobipyramids. Firstly, a capture antibody was immobilized on a 96-well plate with albumin then, different virus concentrations, biotinylated detection antibody and avidin-alkaline phosphatase (avidin-ALP) were added in this sequence. In the end, gold nanoparticles, 4-aminophenyl phosphate (4-APP) and AgNO_3_ were added to the plate. Avidin-ALP catalyzed the decomposition of 4-APP to 4-aminophenol (4-AP), that could reduce Ag^I^ to Ag^0^, directly on AuNP surface. The deposition of silver on AuNP surface led to a change on the reflective index and in the color of the solution. This change was proportional to the thickness of the silver layer which was directly correlated to the virus concentration.

### 3.3. Surface Plasmon Resonance-Based and Fluorescence-Based Sensors

Surface plasmon resonance (SPR) is an optical technique that allows to monitor the growth of ultrathin films close to the sensor interface. Indeed, changes in the refractive index of a metallic surface provoke a variation in the surface-plasmons propagation at the metal-dielectric interface, that can be detected [90]. In the classical SPR-based sensors, the sensor surface is functionalized with a molecule that can interact with a probe. When an accumulation of probe molecules occurs, at the sensor surface, it leads to a change in the refractive index near the sensor surface [91]. Although SPR is a highly sensitive technique, one of its major drawbacks is the inability to use it in low molecular weight biomolecules. The use of nanoparticle tags can remedy this inconvenience through the coupling between the LPSR of the metal nanoparticle and the SPR of the film, together with the increase in the bulk refractive index of the analyte [92]. SPR-based biosensors for microRNA (miRNA) were developed by Hao et al. [93], considering that miRNA has roles in both physiological and pathologic processes and its detection is important for diagnosis, treatment and prognosis of diseases. The protocol was based on the immobilization of biotinylated and thiolated DNA molecular beacon (MB) on a gold film and the use of streptavidin-functionalized AuNRs (Stre-AuNRs) as tag for SPR signal. The plasmonic field extension generated by the approaching of Stre-AuNRs on biotinylated gold film increased the sensitivity of the system and allowed to obtain a LOD 1000-folds lower than that obtained without the use of the Stre-AuNR tag. As for SPR analysis, fluorescence and fluorescence quenching-based methods show ultrahigh sensitivity. When the analyte molecules are close to the metallic nanoparticle, the surface plasmon resonance induces the so called “metal-induced fluorescence quenching” (MIFQ). Hollow porous nanoparticles were used in this contest by Zhao et al. [94] to build a fluorescence quenching-based probe for carcinoembryonic antigen (CEA). CEA is an important cancer marker and its early detection is fundamental for early diagnosis and treatment. AuHPNPs, surface-modified by antibody conjugation, enabled the detection of CEA with a LOD of 1.5 pg/mL.

The use of anisotropic gold-based nanoparticles in bio-sensing is an extremely wide field and many AuNP features can be exploited to have exciting performance and develop high-responsive technique. In Table 1 we summarize the advantages of the very recent advances on this topic.

## 4. Anisotropic Gold Nanoparticles in Therapeutics and Imaging

### 4.1. The Photothermal and Photodynamic Therapy (PTT and PDT)

Photothermal therapy (PTT) is used as a minimal invasive method in cancer therapy, exploiting hyperthermia generated by photothermal agents to kill cancer cells. Thanks to the tissue penetration, near-infrared (NIR) radiation is often employed in photothermal therapy, coupled with light-absorbing materials (known as photothermal agents) that convert NIR light into heat energy. Cancer cells are killed by this hyperthermia when the temperature reaches 42–43 °C, whereas healthy cells tolerate the increase of temperature without further damage. The absorption of NIR light (wavelength 700–850 nm) of anisotropic gold nanoparticles (i.e., nanoshells, nanorods, nanocages and nanourchins) can be exploited for developing high effective photothermal therapy [8,95]. Furthermore, the extent of heat is correlated to the incident excitation power and to the properties of the nanoparticle itself. By optimizing the surface properties, shape and size of the nanoparticle, the plasmonic absorption can be adjusted [96].

Ong et al. used LAT-1 ligands L- and D-dopa as reducing and capping agents in the synthesis of AuNUs to effectively target the large neutral amino acid transporter LAT-1. LAT-1 is a very interesting transporter, further used as a biomarker for imaging and treatment of human malignancies including breast cancer. AuNFs functionalized with L-dopa or D-dopa showed promising results in MCF-7, MDA-MB-231, MDA-MB-468 and MDA-MB-453 cell lines, compared to nontargeting control. Additionally, a successfully combined treatment of chemotherapeutic agents (cisplatin or docetaxel) with L-Dopa@AuNUs, showed that the cell viability was further decreased [97]. Xue et al. [64] reported a one-step synthesis of gold nanoshells (AuNShs) and gold nanocages (AuNCs) by using lanreotide acetate (Lan) as a biotemplate. In vitro NIR irradiation (808 nm, 0.8 W cm^−2^, every 30 s) increased the temperature of AuNCs or AuNShs to 54.3 °C and 46.8 °C. The extent of temperature for AuNCs was much higher than of AuNShs, suggesting better photothermal properties of AuNCs. Furthermore, MTT assays were used to study in vitro antitumor activities of AuNC and AuNShs. The two-prepared anisotropic AuNPs showed significant inhibition on HeLa cells after irradiation at 808 nm, that was 86.26% for AuNCs and 75.56% for AuNShs, which confirmed a strong photothermal effect. In vivo tests on murine model showed remarkable inhibition effects in tumor growth after treatment with AuNCs and AuNShs and NIR irradiation. When mice were injected with AuNCs or AuNShs and irradiated, the tumor surface temperature increased to 48.3 °C (within 5 min, AuNShs) and to over 50 °C (within 3 min, AuNCs). After 12 days the tumor weight decreased significantly in these groups, whereas control groups showed no significant changes. The tumor inhibition rate of AuNCs (86.65%) and AuNShs (72.39%) demonstrated the efficacy in photothermal therapy. Au nanorods, modified with lipoic acid (LA) and chitosan oligosaccharide (COS, introduced to reduce the cytotoxicity caused by CTAB), showed excellent characteristics for photothermal therapy both in in vivo as in in vitro experiments [98]. AuNRs@LA@COS could reach a temperature of 52.6 °C for 5 min of NIR laser irradiation at 2 W/cm^2^. Furthermore, tumor bearing mice treated with such nanosystems (25 µg/mL) and NIR laser irradiation (808 nm) showed an excellent photothermal response as the tumor disappeared completely and normal tissue was reconstructed. To enhance the PTT efficacy, an effective methodology has been introduced by targeting the subcellular entity. The protocol is based on the use of a nuclear localization sequence (NLS) which can help nuclear translocation [99,100], inducing hyperthermia near specific organelles. Chen et al. designed AuNUs coated with cationic NLS for nuclear translocation and with hyaluronic acid (HA) to improve biocompatibility and cellular targeting. These AuNU@NLS@HA nanoplatforms showed very good stability, accurate tumor-targeting, great cellular internalization, good biocompatibility and very good photothermal activity. Moreover, in vivo and in vitro experiments showed promising results and, additionally, inhibition of primary tumors and suppression of metastatic ones were observed [99]. A recent innovation in developing new PTT agents is the use of molecular-imprinted polymers (MIPs). MIPs are artificial synthesized antibodies with high specificity and increased chemical stability respect to substantial antibodies. Yin et al. prepared sialic acid imprinted AuNRs targeting specific cancer cells: these monosaccharide-imprinted AuNRs showed promising photothermal effect of cancer cells without damaging surrounding healthy tissue [101].

**Figure 13:**
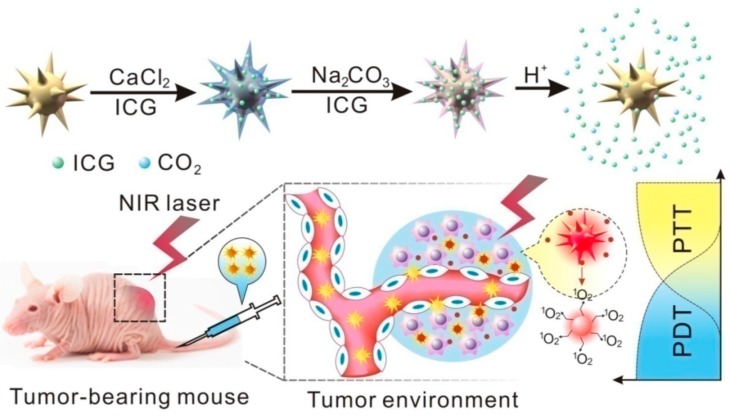
Scheme of construction and release mechanism of pH-trigger release system. Reproduced from [102] with permission from Ivyspring International Publisher.

In the photodynamic therapy (PDT), photosensitizers, used as PDT active agents, are excited by specific wavelength and can generate reactive oxygen species (ROS) that cause cell apoptosis. PDT combined with PTT indicated a promising joint therapy strategy to treat cancer diseases. Combining the use of nanoparticles with specific molecules which generate ROS is an intriguing field, however, drugs can be released too fast from NPs when entering the body thus decreasing the therapeutic effect. Moreover, the intrinsic fluorescence of photosensitizers is quenched from the gold nanoparticle due to Förster resonance energy transfer (FRET) phenomenon, that makes tumor imaging and drug tracing using PDT even more challenging. Therefore, novel methods to load photosensitizers on AuNP are required for an efficient PDT/PTT approach and imaging. For example, indocyanine green (ICG) is a tricarbocyanine dye approved as NIR photosensitizer but, unfortunately, it is characterized by poor stability, fast blood clearance and low quantum yield. Recently, a nanomaterial based on anisotropic gold nanoparticles has been developed with promising results as combined PDT/PTT agent [102]. Anisotropic AuNPs where coated with a layer constituted by CaCO_3_ and ICG, which forms stable aggregates with CaCO_3_ to prevent its fast blood clearance. Furthermore, the CaCO_3_ layer is degraded in acidic medium, therefore ICG can be selectively released in tumor tissue at pH 6.4. In vivo and in vitro experiments using AuNPs@CaCO_3_/ICG nanomaterials after NIR irradiation showed an effective combined antitumor effect; additionally, it was possible to observe the NP biodistribution in the tumor by means of a fluorescence imaging (Figure 13).

### 4.2. The Radio-Therapy

Radiation therapy is still one of the most diffused cancer treatments, but it suffers from the lack of selectivity towards tumor cells which can induce the damage of healthy tissues and organs [103]. Recently, it was shown that targeting nanoparticle-based radio sensitizers can selectively amplify the effect of radiation without damaging healthy surrounding tissues. AuNPs are excellent candidates for the development of this type of sensitizer as they can produce a significant amount of short ranged secondary electrons (i.e., photo electrons, Compton electrons or Auger electrons), which can then further produce reactive oxygen species (ROS) to increase radiation-induced damage [104,105,106,107,108]. Chen et al. studied the synergistic enhancement of radiation sensitization via the interface of Au and titanium oxide within an anisotropic dumbbell-like nanostructure (DAT). The TiO_2_ NPs can absorb ultraviolet light and generate hydroxyl and superoxide radicals, while the Au component interacts with ionizing radiation and forms secondary photons or electrons [106,108]. Secondary photons or electrons can then migrate over the DAT surface to the TiO_2_ part and simplify the production of ROS [106]. The enhancement of radiation sensitization of DATs was validated by in vitro and in vivo experiments with triple-negative breast cancer (TNBC) cells (SUM159). DATs showed a significant increase in ROS production under X-ray irradiation (320 kVp and 12.5 mA with 2 mm Al filter, dose rate was 504.4 cGy/min) compared to titanium dioxide and AuNPs alone. This effect indicates that the energy deposition of low-energy electrons influences the ROS production. In vivo experiments with TNBC-bearing mice showed that the tumor growth was significantly slower in the mice model treated with DATs and X-ray [103].

An overexpression of the enzyme cyclooxygenase-2 (COX-2) is one sign of tumorigenesis and is nowadays an important target for tumor treatment [109,110,111,112]. Small interfering RNA (siRNA) is used for gene suppression but still an effective and reliable siRNA delivery system is needed. Zhu et al. prepared AuNPs modified with 2-amino-2-deoxy-D-Glucose (DG)-polyethylene-glycol (PEG). DG is a glucose analogue able to target by recognizing glucose transporter 1 (GLUT1), overexpressed in cancer cells. GLUT1 can transport DG into cells and furthermore it can act as an indicator of tumorigenesis. AuNPs were further functionalized with lipoic acid (LA), lysine (Lys) and 9-poly-D-argine (9R). 9R was linked via a pH sensitive hydrazone bond and it acted as a carrier for siRNA siCOX-2 into the cytoplasm. In vitro experiments showed an efficient COX-2 suppression by siRNA/9R/DG-AuNPs hydrazone nanomaterials in HepG2 and SGC7901 cells and, additionally, a good photothermal effect [113].

### 4.3. Anisotropic Gold Nanoparticles in Medical Imaging

Visualization of tissues and cell functions are important technique of imaging which can help to detect early stage diseases (i.e., cancer) or to guide therapy for a precise treatment. In the molecular imaging field, many technologies like magnetic resonance imaging, nuclear medicine (i.e., positron emission tomography, PET), optical imaging and ultrasounds find applications [114]. Nanomaterials, with their peculiar properties are excellent candidates to play a crucial role in the development of increasingly efficient and sensible imaging modalities. For example, iron oxide nanoparticles have been developed as good contrast agents for magnetic resonance imaging (MRI) or gold nanoparticles have been proved to be excellent candidate for the next generation of computed tomography contrast agents [33,115]. Anisotropic gold nanoparticles, with their optical features and the possibility to finely tune the LSPR band into the NIR windows, play a further role in the development of imaging modalities. For example, two-photon fluorescence microscopy in the near-infrared spectral window (700–1350 nm) allows tissue penetration at depth non-accessible to conventional confocal microscopy and gives additionally 3-D spatial resolution without damaging surrounding tissue. Chaix et al. synthesized 12 nm anisotropic gold nanoparticles and adsorbed them onto a mesoporous silicon nanoparticles (pSiNPs) of 200 nm, which were further functionalized with mannose to enable endocytosis. Two-photon fluorescence imaging was investigated by pSiNP-SH/AuNPs and pSiNP-SH/AuNPs/Man incubated with human MCF-7 breast cancer cells treatment with Chameleon laser of LSM 780 (Laser input 3 W) at a low power (5% of laser input) under 800 nm extinction wavelength. The porous silicon nanovectors modified with AuNPs and further functionalized with mannose for tumor targeting showed promising results as 2-photon imaging contrast agent. Furthermore, it was shown that pSiNP-SH/AuNP systems provoke oxidative stress in MCF-7 cells under TPE exposure, which can be used in PDT [116].

Compared with traditional imaging techniques, photoacoustic imaging (PAI) combines the excellent contrast of optical imaging and the high penetration depth of ultrasound, which reduces the effect of light scattering on imaging quality and breaks the soft limit of high-resolution optical imaging depth. Consequently, PAI promises to produce high-resolution and highly sensitive tissue images with a depth of 50 mm in in vivo tissue imaging. By coating AuNRs with Ag, the photoacoustic signal decreased, but after mild oxidation method i.e., ferricyanide solution or H_2_O_2_, Ag shells can dissolve and release Ag^+^. Furthermore, the photoacoustic contrast is switching on (silver oxidation) and off (silver deposition) which allows for noninvasive measurement of Ag^+^. These released Ag^+^ ions show a strong bactericidal activity and >99.99% of tested gram-positive and gram-negative bacteria are killed (methicillin resistant Staphylococcus aureus, *E. coli*). In vivo experiments demonstrated that mice treated with Au/AgNRs have much lower bacterial counts in injured tissue that untreated species. Furthermore, Au/AgNRs can release Ag^+^ after stimulation by reactive oxygen species (hydrogen peroxide and peroxynitrite) [117].

A very interesting pH-depending approach to target tumor cells is based on pH-dependent trans-membrane activity, named pH (low) insertion peptides (pHLIPs) [118,119,120]. At low pH, the pHLIPs are activated by protonation of their aspartate or glutamate amino acid residues and, consequently, the hydrophobicity of the peptide chain is increased. Therefore, the pHLIPs can insert in lipid bilayer of tumor cells and form a stable trans-membrane α- helix, allowing nanomaterials to enter easily into the cell. Tian et al. [121] exploiting pHLIP features, by modifying AuNUs with pHLIPs and successfully monitor by CT/PA imaging techniques the tumor targeting in a mouse model. The AuNU@pHLIP showed 1-fold higher cellular internalization with MCF-7 breast tumor cells at pH 6.4 respect to bare AuNUs. In vivo experiments indicated a 3-fold higher tumor accumulation after 24 h and tumor suppression after using NIR-PTT.

**Figure 14:**
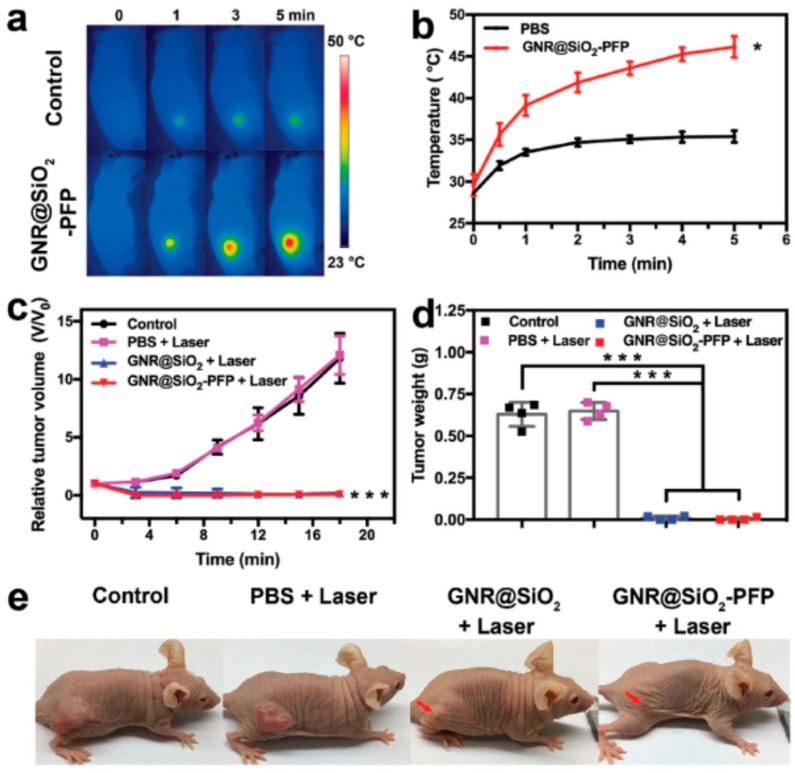
In vivo studies in A375 tumor-bearing mice of GNR@SiO2-PFP nanorattles. (**a**) Thermographic images and (**b**) Temperature change after laser irradiation at 24 h post-injection (* *P* < 0.05). (**c**) Tumor growth after PTT treatment (*** *P* < 0.001) and (**d**) tumor weight of mice on day 18 after treatments (*** *P* < 0.001). (**e**) A375 tumor-bearing mice (red arrow) after treatments. Reproduced from [122] with permission from John Wiley and Sons.

Ultrasound (US) imaging is another imaging approach which finds several applications due to its real-time, noninvasive and easy accessibility. Microbubbles used as US contrast agents can enhance the ultrasonic signal but have a short half-life (<20 min) and a large size (1–8 µm) that hinders their penetration. The design of a proper US contrast agent is, therefore, still an open challenge. In this context, Li et al. designed a perfluoropentane (PFP)-loaded nanorattle constituted by a thin mesoporous silica shell and a gold nanorod core (AuNR@SiO_2_-PFP). Exciting, these nanorattles showed a long blood circulation half-life and were further transformed into microbubbles through NIR irradiation. In vivo studies confirmed the generation of PFP nanobubbles and a combined therapy with PTT showed therapeutic efficacy (Figure 14) [122].

### 4.4. Anisotropic Gold Nanoparticles as Theranostic Agents

Radiation and chemotherapy are the most commonly used therapies in hospitals for cancer treatment. Unfortunately, patients treated with these therapies suffer from many side effects and new approaches are highly desirable. Magnetic hyperthermia, photothermal therapy or photodynamic therapy have fewer side effects and are therefore, under great investigation for further advances. The challenge in this research is to develop innovative agents able to act in the same time as probe to image the damage and as therapeutic agent. Gold nanoparticles, in principle, could act as very promising theranostic agents and the number of publications in this field confirms this idea. Chauhan et al. synthesized AuNRs supported liposome nanohybrid modified with polyethylene glycol, folic acid and loaded with the approved anticancer drug doxorubicin for a chemo/photothermal therapy. The AuNR-liposome nanohybrid induced an increasing of temperature of 43 °C in 5 min after NIR treatment (0.2 mg/mL, 650 nW). Drug release studies confirmed the successful drug release in acidic environment, whereas almost no doxorubicin was released from the AuNR-liposome nanohybrid in neutral pH environmental (4% of DOX release). DOX-loaded and PEG-FA modified nanohybrid induce after NIR exposure of 5 min (100 µL in 200 µg/mL concentration) more than 90% cell death. Furthermore, these modified AuNRs showed to be very well visualized via computed tomography [123]. Folate-targeted gold nanorods are studied in osteosarcoma therapy as theranostic agent by Volsi et al. The group prepared two novel folate targeted gold nanorods systems with two versatile and cyto-compatible polymers (inulin and α,β-poly(N-2-hydroxyethyl)-DL-aspartamide PHEA derivatives) functionalized with folic acid. Both nanosystems (AuNRs@INU-LA-PEG-FA and AuNRs@PHEA-EDA-FA) were further studied as anti-cancer drug delivery system, carrying nutlin-3. Both AuNRs systems showed physiochemical stability and specific drug release, especially PHEA-EDA-FA coated AuNRs, that indicated a pH-dependent drug release. In vitro studies displayed that both systems act as effective drug delivery nanostructures, hyperthermia agents and imaging contrast agents [124]. Theranostic nanoagents can be used as a combination of cancer treatment and imaging for synergistically improved treatment with the use of either therapy or imaging only. Zhang et al., proposed a strategy for the fabrication of flower-like gold nanorod/polydopamine bowl (AuNR/PDA bowl) with a spadix-bract nanostructure. Additionally, these flower-like NPs were functionalized with 1-dodecanethiol (DDT), which operated as storage space and passage for hydrophobic anticancer drug 10-hydroxycamptothecin (HPTC). Then, the surface of the PDA bowl (bract) was also decorated with doxorubicin. The flower-like DT-AuNR/PDA bowl spadix bract simplifies the solubility problem of HPTC and also prevent degradation. DT-AuNR/PDA bowl spadix-bract nanosystems showed high loading capacity for HCPT and DOX and photothermal efficiency. In vivo experiments with dual-drug-loaded DT-AuNR/PDA bowl spadix-bract NPs combined with NIR laser irradiation indicated better treatments than solo-drug-loaded or dual-drug-loaded groups [125].

Hyaluronic acid (HA) is a natural negatively charged polysaccharide with pharmaceutical potential. Most importantly, HA can interact with CD44 receptor, which is overexpressed in breast cancer [126,127]. HA-functionalized AuNPs (i.e., nanosphere [127], nanostar [128], nanocage [129]) target cancer cells by passive enhanced permeability and retention (EPR) effect and by active HA-mediated targeting. Xu et al. reported the development of a pH and NIR dual-responsive nanoplatform AuNRs-HA-FA-DOX for photothermal chemotherapy, loading the chemotherapeutic drug via a pH sensitive hydrazone bond. In vitro experiments on MCF-7 cells showed that treatment of these cells with GNRs-HA-FA-DOX and NIR irradiation induced efficiently cell apoptosis. In vivo studies on mice born tumors treated with AuNR-HA-DOX and AuNR-HA-FA-DOX and further irradiated with 808 nm laser at 1.5 W/cm^2^ showed that an increase in temperature to 45.9 °C and 48.6 °C after 1 min was achieved, stating a therapeutic efficacy [130]. Recently, rod-like Janus AuNR@PMO (PMO = periodic mesoporous organosilica) nanoparticles showed high efficacy in cancer chemo-photothermal combination therapy, by exploiting the PMO capability as drug delivery system. AuNRs with a LSPR absorption peak at 915 nm were used to produce AuNR@PMO by means of a further modification with octadecyltrimethoxysilane (C_18_TMS) and PEG-grafted poly(maleic anhydride-alt-1-octadecene). The approved chemotherapeutic drug doxorubicin was used in this study as a model for drug delivery system. UV-Vis analysis confirmed the successful loading of DOX within the nanostructures (mass ratio of DOX to AuNR@PMO-PEG = 1.3:1), whereas a moderate DOX loading was used for further investigations (mass ratio of DOX to AuNR@PMO-PEG = 0.6:1). Previous studies showed that DOX might be released violently upon photothermal stimulations [131,132]. In vitro experiments of a combined chemotherapy and photothermal therapy on 4T1 murine breast cancer cells showed enhanced efficacy due to this combined therapy. For control, two further experiments were conducted and cells were treated with AuNR@PMO-PEG/DOX but not irradiated with NIR light and cells were incubated with AuNR@PMO-PEG for the only PTT: in both cases the outcomes showed a lower therapeutic efficacy. Thanks to the laser irradiation, the cellular uptake of nanoparticles may be increased and the drug doxorubicin released from the nanocarrier, as confirmed by fluorescence imaging in vitro, confirming the potentiality of dual therapy [133].

Recently, it was shown, that photothermal treatments trigger cancer cells death by a programmed apoptosis with activation by the caspase-3 pathway [134]. Wang et al. created AuNUs with photothermal properties and additionally, a self-theranostic feedback based on the switch-on fluorescence for caspase-3 imaging features. The AuNUs were functionalized with FA for targeting tumors, a caspase-3 responsive peptide linker (DEVD, Asp-Glu-Val-Asp), and a NIR fluorescent dye (atto 655). These AuNUs served as fluorescent quencher, which is activated by cell apoptosis induced by the caspase-3 pathway due to the treatment with NIR light. Moreover, these functionalized AuNUs showed a photothermal therapeutic effect as self-feedback imaging of caspase-3 [135].

An et al. synthesized a novel multifunctional nanoprobe for PAI and CT dual imaging, combining in the same complex nanostructure even a chemotherapeutic agent. Firstly, AuNUs were coated with a mesoporous silica shell (GMS), then thermo-sensible lipid bilayers formed by folic acid (FA)-modified poly(ethylene glycol) (PEG)-phospholipid, dipalmitoyl phosphatidylcholine and distearoyl phosphatidyl choline mixture (SLB-FA) were used to cover the silica shell. Finally, the system was loaded with doxorubicin. In vivo and in vitro studies showed a good targeting effect of GMS/DOX@SLB-FA and furthermore, the combined therapy approaches (chemotherapy and phototherapy) enhanced the treatment. Doxorubicin release from mesoporous channels which were sealed with thermosensitive phospholipid membrane to control the release by NIR laser provided exciting and promising results [136].

The use of anisotropic gold-based nanoparticles in theranostics is very exciting and the promises for future damage early detection and on-site therapy pushes the research toward continuous attempts and innovation. In Table 2 we summarize the very recent employment of AuNPs in the field.

## 5. Conclusions

Anisotropic gold nanoparticles represent one of the most exciting and promising type of gold nanomaterials. Their LSPR tunability and photophysical properties make them the ideal candidates for many developments in a wide range of bio-applications. In this review we collected the very recent advances related to the synthesis and bio-applications of anisotropic gold nanoparticles. New synthetical approaches, developed with the final goals to produce anisotropic nanomaterial in a reliable, controlled and safe way, are fundamental requirements for boosting a strong use of gold-based nanoparticles in clinics. Besides the protocol to produce AuNPs, a reliable surface functionalization represents a second critical point. The bio-compatibility, the active targeting and the ability of the nanoparticles to circulate in the blood until the desired target, without being processed by the macrophages, depend on the type of their functionalization. When AuNPs are used, in vivo or in biological fluids, proteins adsorb on gold surfaces forming the so-called protein-corona, which can be envisaged as a completely new entity respect to the synthesized one. Once formed, protein-corona increase the overall nanoparticle size, cover the eventual target moiety and let the nanoparticles be processed by macrophages. Therefore, new and specific ligands to overcome protein-corona formation should be designed, in order to have and manipulate nanoparticles able to do exactly what they are projected for. Finally, the third critical point which needs to be carefully addressed is the implementation of nanotoxicology studies, which are urgent and fundamental for a concrete clinical translation of these nanoparticles so that they can fulfil their promised potential. 

## Figures and Tables

**Table 1:** Overview of anisotropic gold nanosystems used in bio-sensing.

NP Shape	Sensing Technique	Scope	References
AuNUs	SERS	Targeted diagnosis of immunomarker programmed death ligand 1 (PD-L1) and detection of epidermal growth factor receptor (EGFR) in breast cancer tumors in vivo.	[76]
AuNUs	SERS	Quantitative measurement of extracellular protein kinase A (PKA) activity as cancer biomarker.	[77]
AuNRs	SERS	Detection of intercellular adhesion molecule-1 (ICAM-1) in macrophages and in endothelial cells as indicators for clinical diagnosis of diseases correlated to inflammation.	[78]
AuNUs	SERS	Protein, lipid, nucleic acid, amino acids and carbohydrates to detect differentiation of BMSCs (bone marrow mesenchymal stem cells).	[81]
AuNUs	SERS	Development of a lateral flow strip for neuron-specific enolase (a traumatic brain injury protein biomarker) detection in blood plasma.	[79]
AuNRs	SERS	Circulating tumor cells detection.	[80]
AuNUs	SERS	Enterovirus 71 (EV71) detection.	[82]
AuNUs	SERS	Semi-quantitative methodology to detect carbapenemase activity in patients.	[83]
AuNRs	Colorimetry	Poly (ADP-ribose) measure, to detect poly(ADP-ribose) polymerase-1 (PARP-1) activity.	[87]
AuNUs	Colorimetry	Influenza virus detection.	[89]
AuNR	SPR	Design of a sensitive microRNA biosensor for disease diagnosis, treatment and prognosis.	[93]
AuNCs	Fluorescence quenching	Synthesis of a novel carcinoembryonic antigen (a cancer biomarker) probe.	[94]

**Table 2:** Overview of anisotropic gold nanosystem for therapeutics and imaging.

Nanoparticle	NP Size	Outcome	Cell Line	Ref.
AuNCs or AuNShs	Diameter: 120 nm/150 nm	EPR targeting effect and PTT efficiency	HeLa cells	[64]
AuNRs-LA-COS	Length: 26 ± 3.1 nm Diameter: 6.8 ± 1.7 nm	PTT efficiency	MDA-MB-231	[98]
AuNU-NLS@HA	Diameter: 93.2 nm	PTT efficiency	NIH3T3, MCF-7, 4T1	[99]
SA-imprinted AuNRs@SiO_2_	Length: 40 nm Width: 10 nm	Effective cancer-cell-targeting and PTT efficiency	HepG-2, L-02	[101]
AuNUs	Diameter: 46, 70 and 90 nm	PTT and chemotherapeutic efficiency	MCF-7, MDA-MB-231, MDA-MB-468, MDA-MB-453, MCF-10A	[97]
AuNU@CaCO_3_/ICG	Diameter: 96 ± 7.5 nm	PDT/PTT efficiency	MGC803	[102]
Au@TiO_2_ (DATs)	Diameter: 70.1 ± 4.9 nm	PDT efficiency	SUM159	[103]
AuNUs, 9R-AuNUs, 9R/DG- AuNUs, 9R/DG-AuNUs hydrazone	Diameter: 72.5 ± 3.2, 77.8 ± 9.7, 230.7 ± 8.6, and 210.5 ± 10.3 nm	Delivery of siCOX-2; PTT efficiency	HepG2, SGC7901	[113]
pSiNP-SH/AuNP pSiNP-SH/AuNP/Man	Diameter: 270 nm Diameter: 328 nm	Oxidative stress; 2-photon imaging	MCF-7	[116]
Au/Ag hybrid nanoparticles	Width: 12−14 nm Length: 50 nm	PA imaging; antibacterial activity	SKOV3	[117]
AuNU-pHLIP	Diameter: 60 nm	CT/PA-guided PTT efficiency	MCF-7	[121]
AuNR@SiO_2_-PFP	Length: 52.24 ± 17.21 nm Width: 16.81 ± 3.97 nm	US/PA imaging guided PTT efficiency	A375	[122]
AuNR-Lipos	Length: ~27 nm Width: ~9 nm	synergistic chemo/PTT	MDA-MB-231	[123]
AuNRs@INU-LA- PEG-FA and AuNRs@PHEA-EDA-FA	Length: ~70 nm	synergistic chemo/PTT	U2OS	[124]
DT-AuNR/PDA bowl spadix-bract NP	Diameter: 90 ± 13 nm	Chemo/PTT therapy CT/PA imaging	Hep-G2, HeLa, MCF-7	[125]
GNRs-HA-FA-DOX	Diameter: 70.9 ± 1.4 nm	Chemo/PTT therapy	MCF-7	[130]
AuNR@PMO-PEG/DOX AuNR@PMO-PEG	Length: 578.5 ± 42.6 nm Diameter: 112.4 ± 12.8 nm	Drug delivery	4T1 murine breast cancer cells	[133]
AuNU@probe	Diameter: 35 nm	PTT efficacy	U87-MG	[135]
GMS/DOX@SLB-FA	Diameter: 150 nm	Drug delivery	HeLa, A549	[136]

Abbreviations. LA: lipoic acid; COS: chitosan oligosaccharide; NLS: nuclear localization sequence; SA: sialic acid; LAT-1: Large neutral amino acid transporter; ICG: indocyanine green; DATs: dumbbell-like nanostructure; DG: 2-amino-2-deoxy-d-Glucose; 9R: 9-poly-d-argine; Man: mannose; pHLIPs: pH (low) insertion peptides; PFP: perfluoropentane; INU: inulin; PHEA: α,β-poly(N-2-hydroxyethyl)-DL-aspartamide; EDA: Ethylenediamine; FA: Folic acid; DT: 1-dodecanethiol; PDA: Polydopamine; HA: hyaluronic acid; DOX: doxorubicin; PMO Mesoporous organosilica; GMS: mesoporous silica shell; SLB-FA supported lipid bilayer-folic acid.
